# Dietary supplementation with pure or plant-derived phytochemicals alters growth performance, lipid profiles, hepatic gene expression, and intestinal health in broiler chickens

**DOI:** 10.1016/j.japr.2026.100683

**Published:** 2026-02-16

**Authors:** Samar A. Tolba, Evan A. Chrisler, Jake B. Hermanson, Jordyn Z. Figur, Sean M. Baker, Richard S. Reiner, Rajai H. Atalla, Mark P. Richards, Vanessa A. Leone

**Affiliations:** a Department of Animal & Dairy Sciences, University of Wisconsin-Madison, Madison, WI 53706, USA; b Department of Nutrition and Clinical Nutrition, Faculty of Veterinary Medicine, Zagazig University, Zagazig 44511, Egypt; c Department of Nutritional Sciences, University of Wisconsin-Madison, Madison, WI 53706, USA; d Department of Food Science, University of Wisconsin-Madison, Madison, WI 53706; e Cellulose Sciences International, Madison, WI 53719, USA

**Keywords:** Ferulic acid, Redox gene expression, Intestine, Microbiota, Broiler

## Abstract

This study evaluated pure ferulic acid (**FA**) or a FA-rich corn pericarp extract (**CPE**) dietary supplementation on broiler growth performance and associated physiological and microbial responses. Ross 708 d-old male chicks (*n* = 300) were randomly assigned to three corn-soybean meal diets: no additive (Control), 100 mg/kg FA, or 600 mg/kg CPE, standardized to provide 100 mg/kg FA equivalents. From 0–21 d, birds were reared in battery cages (5 birds/pen; 20 pens/treatment) with a subset transferred to floor pens (10 birds/pen; 3 pens/treatment) from 22 to 42 d. No overall differences in growth performance were observed from 0 to 21 d; however, differences within wk were detected. At 7 d, CPE increased BW, body weight gain (**BWG**), and **FI** compared to FA, while BWG was reduced at 14 d. FA increased BW at 35 and 42 d along with BWG from 22 to 42 d compared to CPE. CPE increased intestinal weights at 42 d and altered duodenal morphology at 21 d, including decreased villus height (**VH**), increased crypt depth (**CD**), and reduced VH/CD ratio. At 42 d, both FA and CPE upregulated hepatic *GPX1* expression and modulated cytokine gene expression. Age primarily influenced microbial diversity; however, FA and CPE promoted early enrichment of beneficial taxa at 7 d. Overall, age primarily drove physiological and microbial changes; however, FA and CPE differentially influenced growth, intestinal development, hepatic function, and gut microbiota in an age-dependent manner. These findings support potential for FA-based additives to impact broiler performance, with associated effects on gut health and physiology.

## Description of problem

Antibiotic growth promoters (**AGP**) are used globally as feed additives in the poultry industry to prevent diseases and improve performance outcomes (Gadde et al., 2017). However, over the past two decades, concerns surrounding antibiotic stewardship and antimicrobial resistance have led to strong regulatory action in major production regions, including a ban on AGPs in the European Union since 2006 and the phase-out of medically important AGPs in the United States (Castanon, 2007; Armbruster and Roberts, 2018). Safe, cost-effective, and natural AGP alternatives are in high demand for the poultry industry. Botanical products, including plant-derived phytogenic feed additives (**PFA**), contain various bioactive compounds that possess beneficial effects, including antioxidant, antimicrobial, immunomodulatory, and anti-inflammatory activities, without negatively impacting growth performance, and can therefore be utilized as alternatives to AGP in poultry production (Hernández et al., 2004; Lillehoj et al., 2018; Kuralkar and Kuralkar, 2021; Abdelli et al., 2021).

Globally, corn is the most common dietary feed ingredient, containing significant amounts of essential nutrients and bioactive compounds that elicit desirable growth outcomes and health benefits (Siyuan et al., 2018). The three major components of corn kernel (endosperm, germ, and bran) each have unique phytochemical profiles (Salinas-Moreno et al., 2017). Corn bran and germ fractions contain high phytochemical levels, including several phenolic acids, including FA (4-hydroxy-3-methoxycinnamic acid) (Adom and Liu, 2002). Whole corn contains ~900 μmol total FA /100 g of grain, where ~98.9% is bound (mostly in the pericarp), 1% is in soluble conjugate form, and only 0.1% is found in the free form (primarily located in the germ) (Dewanto et al., 2002; Adom and Liu, 2002; Salinas-Moreno et al., 2017). Therefore, various strategies are needed to release bound FA, which can enhance total antioxidant activity, optimizing the nutritional value of corn (Dewanto et al., 2002).

Several recent studies show FA has a wide range of biological activities, including antibacterial, antioxidant, anti-inflammatory, antithrombotic, and anticancer properties (Shi et al., 2016; Choi et al., 2018; Zheng et al., 2019; Chowdhury et al., 2019; Rehman et al., 2019). Dietary inclusion of FA has been studied in a variety of different species, including rodents, pigs, fish, and ducks. For instance, FA dietary inclusion elevated hepatic reduced glutathione (**GSH**), enhanced antioxidant activity, and lowered malondialdehyde levels in rodent models of liver oxidative stress (Balasubashini et al., 2004; Kim et al., 2011). Likewise, dietary inclusion of FA enhanced liver antioxidant capacity in a dose-dependent manner and significantly increased hepatic mRNA transcript abundance of lipase E, hormone-sensitive type (***LIPE***, which encodes hormone-sensitive lipase), carnitine palmitoyltransferase-1b (***CPT1B****)*, and peroxisome proliferator-activated receptor α (***PPARA***) in weaned piglets (Wang et al., 2020). Furthermore, FA supplementation at varying inclusion levels improved growth performance, intestinal morphology, antioxidative and immunological capabilities, and altered gut microbiota composition during the grower phase of Linwu ducks (Liu et al., 2021b), and improved gut health when incorporated into fish diets at different concentrations (Fu et al., 2022). To the best of our knowledge, limited research has examined dietary FA in broiler chickens on performance traits. For example, Tang et al. (2023) demonstrated that dietary FA reduced lipopolysaccharide (**LPS**)-induced intestinal damage in broilers by enhancing antioxidant capacity, preserving intestinal morphology and barrier integrity, and modulating gut microbiota composition. Additionally, Du et al. (2022) showed that dietary FA improved broiler growth performance during the starter phase by enhancing digestive enzyme activities, antioxidant potential, and immune status. Collectively, these studies highlight the promising potential of FA to improve production outcomes across a wide array of species. However, pure FA is costly, limiting its broad-scale utility as a dietary supplement in agricultural settings (Overhage et al., 2003; Martău et al., 2021).

The constraints of using pure FA have prompted interest in using FA-rich botanical products as economical alternatives. In this study, we evaluated the effects of pure FA versus a FA-rich corn pericarp extract (**CPE**) derived from a waste stream of the corn ethanol industry on broiler performance. We hypothesized that pure FA and CPE would exhibit comparable capacity to improve growth performance, carcass traits, as well as promote intestinal and liver health parameters through modulation in antioxidant and anti-inflammatory capacity relative to an unsupplemented control diet. Secondarily, we anticipated CPE would induce distinct changes in gut microbiota composition relative to both pure FA and control diets.

## Materials and methods

### Ethics statement

All procedures for this trial were approved by the University of Wisconsin-Madison Institutional Animal Care and Use Committee (IACUC A005392-R03).

### Experimental design, birds, diets, and management

Hatchling Ross-708 male cockerels (*n* = 300) were purchased from Welp Hatchery (Bancroft, IA). Chicks were weighed upon arrival, wing banded, and allotted to one of three experimental dietary treatments, consisting of a corn-soybean meal basal diet with no additive (Control), 100 mg/kg feed of pure trans-FA, and 600 mg/kg feed of CPE, standardized to provide 100 mg/kg feed of FA equivalents (Cellulose Sciences International, Madison, WI), in a complete randomized design. The FA was purchased from Sigma-Aldrich (St. Louis, MO, USA; #128708; CAS #537-98-4; ≥99% purity). The FA equivalents in CPE were determined via the Folin-Ciocalteu method as previously described (Ainsworth and Gillespie, 2007). Diets were formulated according to the Ross-708 broiler feed specifications (Aviagen, 2022). The basal diet was composed primarily of corn and soybean meal, supplemented with vegetable oil, monocalcium phosphate, and a vitamin–mineral premix (Table 1). Starter, grower, and finisher diets were provided from 0–10 d, 11–24 d, and 25–42 d of age, respectively. The experiment lasted 42 d, with fresh water and mash feed provided *ad libitum* throughout the study. From 0–21 d of age, birds were housed in battery cages at a density of 100 birds per treatment (5 chicks/cage, 20 cages/treatment). At 21 d, one bird per cage was sampled for intermediate measurements (10 birds/treatment). From the remaining cages not used for sampling, six per treatment were randomly selected and combined in pairs (5 birds + 5 birds) which were transferred intact to form 3 floor pens per treatment (10 birds/pen) from 22 to 42 d. All transfers were conducted on the same d. The floor pens were managed identically across treatments, with uniform litter type, feeders, and drinkers. At 42 d, three or four birds per pen were sampled for endpoint measurements (10 birds/treatment). Management conditions throughout the experiment were consistent with the Ross broiler management guide (Aviagen, 2025). For the first three d, the lighting system was set to 23:1 h light:dark, then to 20:4 h light:dark until the end of the study.

### Growth performance and mortality

Birds were weighted upon arrival and every wk to determine live BW and body weight gain (**BWG**). Birds that died within the first 48 h of the experimental period (one bird in the Control group and two birds in the FA group) were considered non-experimental losses and were immediately replaced with backup birds of similar age and BW to maintain stocking density. Mortality occurring after the first 48 h was recorded daily, and birds were not replaced. Performance and mortality data were analyzed based on the number of birds per pen following the initial replacement period. Mortality was calculated at the pen level as the number of dead birds divided by the initial number of birds in the pen, multiplied by 100. For each replicate cage/pen, FI was calculated as the difference between the initial feed offered and remaining feed weight. The FCR was calculated at the cage/pen level as the ratio of total FI (g) to BWG (g), and was corrected for mortality by including the BW of birds that died during the experimental period, using the weight measured at death, as previously described (Amer et al., 2021).

### Sample collection, blood biochemical analysis, and carcass traits

Fresh excreta were collected using sterilized spoons and forceps from 10 battery cages/treatment at 7, 14, and 21 d, and three floor pens/treatment at 35 and 42 d, being careful to avoid cecal droppings. Samples were placed in sterile screw-cap tubes and stored at −20°C for subsequent 16S rRNA gene amplicon sequencing. At 21 and 42 d, non-fasted birds (*n* = 10/treatment) were chosen randomly from each treatment and euthanized via CO_2_ asphyxiation. Blood samples were collected via cardiac puncture into heparinized tubes. Plasma was separated via centrifugation at 3000 g for 15 min at 4°C (Thermo Scientific Sorvall ST 16R, Waltham, MA), followed by storage at −80°C until further analysis. Total cholesterol (**TC**), total triglyceride (**TG**), and non-esterified fatty acid (**NEFA**) concentrations in plasma were measured using commercial kits (FUJIFILM Wako Chemicals, Richmond, VA) following the manufacturer’s instructions. Dressing percentages of liver, intestine, spleen, gizzard, heart, and Bursa of Fabricius were calculated as a proportion of live BW (Amer et al., 2021). Liver sections were immediately snap-frozen in liquid nitrogen and stored at −80°C until further analysis. Approximately two cm sections of the small intestine (**SI**; duodenum, jejunum, ileum) and cecum were collected and placed in 10% neutral buffered formalin for 24 h, followed by transfer to 70% ethanol for subsequent histopathological examination (Giannenas et al., 2010).

At 21 and 42 d, cecal contents from five birds/treatment were collected into RNase/DNase-free screw-cap tubes (Thermo Fisher Scientific, Waltham, MA, USA) and snap-frozen in liquid nitrogen, followed by storage at −80°C for subsequent cecal 16S rRNA gene amplicon sequencing analysis.

### Intestinal histomorphology

Fixed tissue samples from duodenum, jejunum, ileum, and cecum were paraffin-embedded, and 5 μm sections were cut via a microtome and stained with hematoxylin and eosin (**H&E**) by the University of Wisconsin-Madison Translation Research Initiatives in Pathology laboratory. Intestinal sections were imaged on a BZ-X800 All-in-One Keyence Microscope (Keyence, Itasca, IL) at 100x and 200x magnification (BZ-PA10, Plan Apochromat, WD 4 mm). Morphometric measurements (μm), including villus height (**VH**) (from the tip of the villus to the villus-crypt junction), villus width (**VW**) (measured in the median region of each villus), and crypt depth (**CD**) (estimated from the villus-crypt junction to the crypt distal limit), were obtained from intestinal sections collected from 5 to 10 birds per treatment and age. Measurements were performed using ImageJ 1.54 (National Institute of Health, USA), and data are presented as means ± SEM.

### Quantitative real-time PCR

Total mRNA was isolated and purified via phenol:chloroform extraction from ~20 mg of liver tissue using TRIzol reagent (Life Technologies, Carlsbad, CA), followed by reverse transcription using the iScript^™^ gDNA clear cDNA Synthesis Kit (Bio-Rad Laboratories, Inc., Hercules, CA) following the manufacturer’s instructions. Quantitative Real-Time PCR (**RT-qPCR**) was used to quantify expression levels of mRNA transcripts of key antioxidant and cytokine genes on a Bio-Rad CFX384 Real-Time PCR Detection System (see Table S1 for primer sequences). The optimized RT-qPCR cycling conditions were as follows: initial denaturation at 95°C for 5 min, followed by 45 cycles of denaturation at 95°C for 10 s, annealing at 58°C for 20 s, and extension at 60°C for 30 s, with data acquisition occurring at the end of each extension step. Relative gene expression was determined using the 2^−ΔΔCt^ method, with Glyceraldehyde 3-phosphate dehydrogenase (***GAPDH***) as the housekeeping gene and expression levels normalized to Control birds (set as 1) (Livak and Schmittgen, 2001).

### DNA extraction, 16S rRNA gene amplicon sequencing, and bioinformatic analyses

DNA extraction was performed as previously described (Wang et al., 2009) from a total of 138 samples for both fecal excreta collected at the cage/pen-level (*n* = 108; 10 cages/treatment at 7, 14, and 21 d and three pens/treatment at 35 and 42 d) and cecal contents collected from individual birds following euthanasia (*n* = 30; 5 birds/treatment at 21 and 42 d). DNA concentration was determined using a NanoDrop^™^ OneC Microvolume UV Vis Spectrophotometer (Thermo Fisher Scientific, Waltham, MA) and diluted to 10 ng/μL in Ambion^™^ Nuclease-Free Water (Thermo Fisher Scientific, Waltham, MA). Library preparation and sequencing of the V4 region of the 16S rRNA gene were performed utilizing primers as previously described (Kozich et al., 2013). A total of 2,219,333 raw reads (2 × 250 bp) were obtained, with an average value of 16,200 reads/sample. One fecal sample from the Control group at 7 d failed to amplify and was excluded from further analysis. Paired-end demultiplexed reads were imported and filtered utilizing Quantitative Insights Into Microbial Ecology (**QIIME2**, v2023.5). Divisive amplicon denoising algorithm (**DADA2**) was used to filter and denoise the imported demultiplexed sequences (via q2-dada2), where a total of 2,209, 045 reads passed quality and filter checks with an average of 16,124.416 reads/sample.

Fecal and cecal Amplicon Sequencing Variants (**ASVs**) were assigned via Multiple Alignment Using Fast Fourier Transform (**MAFFT**), and a rooted phylogenetic tree was created using FastTree2 (via q2-phylogeny). A classifier pre-trained on the Silva 138 release for the 515F/806R region of the 16S rRNA gene (silva-138-99-515-806-nb-classifier.qza) was used as a reference database (Price et al., 2010; Quast et al., 2013). Taxonomy-based quality filtering via q2-taxa was performed to remove mitochondria, archaea, chloroplast, and eukarya ASVs prior to alpha and beta diversity calculations and downstream statistical analyses. Alpha diversity metrics (Observed, Pielou’s Evenness, and Shannon Diversity), as well as beta diversity metrics (Bray-Curtis Dissimilarity and Weighted and Unweighted UniFrac Distances) were determined in RStudio (Version 2024.04.2024 “Puppy Cup”) utilizing phyloseq package V1.42.0.

### Statistical analysis

Broilers were moved from battery cages to floor pens at 22 d due to facility constraints. Consequently, comparisons of data collected early (0–21 d) and later (22–42 d) should be interpreted within each phase, as potential effects of the housing transition cannot be disentangled from diet or age effects.

Data were analyzed via one-way ANOVA using GraphPad Prism version 10.1.1 (GraphPad Software Inc., San Diego, CA). A Tukey’s Honest Significant Difference Test was performed to assess differences between mean values for growth performance, carcass traits, intestinal parameters, plasma lipids, and hepatic inflammatory and redox transcriptional profiles. A nonparametric Kruskal-Wallis with Dunn’s *post hoc* test was used to analyze mortality, given the discrete and non-normal distribution of the data. For all analyses, the pooled standard error of the mean (**SEM**) is presented unless stated otherwise, and significance was set at *P* < 0.05.

All diversity and taxonomic relative abundance metrics were analyzed via two-way ANOVA to test for main effects of age, diet, and age x diet interactions. Where significant interactions were detected, pairwise Wilcoxon Rank sum tests were performed across age and dietary treatments. To assess differences in community membership, permutational multivariate analysis of variance (**PERMANOVA**) was conducted using the adonis2 function (R package Vegan, version 2.6–4) on Bray-Curtis and Unifrac distance matrices, with diet and age as the variables of interest (Mandal et al., 2015).

Multivariable associations between diet and microbial features were tested using Multivariate Association with Linear Models 2 (**MaAsLin2**, version 1.12.0) in R v4.4.0, with the Control group set as the reference level (Mallick et al., 2021). Any ASV level association with a false discovery rate (FDR)-correlated *q*-value of < 0.25 was considered statistically significant. For enriched taxa identified via MaAsLin2, a two-way ANOVA was conducted to test for main effects of age, diet, and age x diet interactions. Where significant interactions were detected, pairwise Wilcoxon Rank sum tests were performed across age and diet treatments. If only main effects were detected, MaAsLin2 statistics are presented within age (d).

All 16S rRNA gene amplicon sequencing analyses and figures were generated in R Studio (Version 2024.04.2024 “Puppy Cup”) using R v4.4.0.

## Results and discussion

### Effects of pure FA and FA-rich CPE dietary supplementation on broiler growth performance

We tested whether dietary supplementation with pure FA or FA-rich CPE would elicit similar effects on broiler growth performance relative to the unsupplemented Control diet. Overall mortality was low throughout the experiment, totaling 7, 2, and 5 birds in the Control, FA, and CPE groups, respectively, with no significant differences between treatments from 0–21 d or 22–42 d (Table 2).

No significant differences (*P* > 0.05) were observed in growth performance metrics between diets during the 0–21 d period (Table 2). During the early phase (0–7 d), birds fed CPE-supplemented diet showed significantly greater BW, BWG, and FI relative to birds fed pure FA (*P* < 0.05). However, BWG of both supplemented groups were comparable to the Control (*P* > 0.05; Table 2). Interestingly, while no difference in BW was observed between the CPE-supplemented group and the Control (*P* > 0.05), CPE-fed birds showed higher FI (*P* < 0.05; Table 2). From 14–21 d, birds fed CPE had decreased FI relative to both Control and FA (*P* < 0.05). In the later phase (22–42 d), FA supplementation continuously promoted greater BWG than CPE (*P* < 0.05), although FA remained similar to the Control. Birds fed FA exhibited increased BW relative to CPE-fed birds at 35 and 42 d (*P* < 0.05), although both were comparable to Control (Table 2). Birds fed CPE-supplemented diet exhibited the lowest BWG on 28 d relative to FA and on 35 d relative to both FA and Control groups (*P* < 0.05), with no significant changes observed on 42 d. These findings suggest that growth responses to pure FA and FA-rich CPE vary over time. Supplementation with CPE enhanced early growth performance (0–7 d), which was not sustained, however, FA appeared to enhance performance at later stages.

Genetic selection for fast growth rates in broilers leads to increased levels of oxidative stress as birds age, driven by rapid cell proliferation and accumulation of reactive oxygen species (**ROS**) (Surai et al., 2019). The nutritional status of broilers during the starter phase is critical for immune and gastrointestinal tract development and can affect overall health throughout subsequent growth periods (Ravindran and Abdollahi, 2021). During this early period, chicks rely heavily on absorbable nutrients to cope with challenges in their environment (Noy and Sklan, 2001). Our observation that dietary inclusion of CPE resulted in improved growth performance during the first wk post-hatch may suggest its components are more readily absorbed during this critical early life stage, positively impacting BWG and FI.

Further, our findings align with those of Cross et al. (2011), who reported significantly increased BW and BWG at 7 d of age in broilers supplemented with a phytochemical-rich *Allium sativum* L. powder, with diminishing effects after this period. Similarly, *Hibiscus sabdariffa* L. extract supplementation reduced FI and improved FCR during the starter phase in a dose-dependent manner, with limited effects observed during the grower and finisher phases (Amer et al., 2022). Conversely, broilers supplemented with lotus leaf (*Nelumbo nucifera Gaertn*) extract exhibited increased average daily gain from 7 to 21 d dependent on inclusion level (Cheng et al., 2021). The transient nature of these responses may relate to developmental changes in intestinal maturation, enzymatic activities, and nutrient absorption capacity as birds age, which can alter the efficacy of bioactive phytochemicals (Abdelli et al., 2021). The current study indicates that FA supplementation resulted in more stable BWG compared to CPE during later growth phases, implying that purified FA may support antioxidant and metabolic regulation beyond early development, while CPE responses may be influenced by the bioavailability and composition of its complex phytochemical structure (Abdelli et al., 2021). To our knowledge, no prior study has examined the effect of dietary CPE on broiler performance. Due to variations in phytochemical sources and inclusion levels examined, direct comparisons across studies are challenging, highlighting the need for additional research to elucidate the stage-specific impact of dietary CPE as well as optimal dosing.

### Effects of pure FA or FA-rich CPE dietary supplementation on carcass traits and intestinal histomorphology

Dressing percentage and relative organ weights at 21 and 42 d are shown in (Table 3). At 21 d, CPE supplementation had no effect on carcass traits. By 42 d, birds supplemented with CPE exhibited higher intestinal dressing percentages compared to both Control and FA-fed birds (*P* < 0.050), while no differences (*P* > 0.050) were evident at either time point for liver, gizzard, heart, spleen, or Bursa of Fabricius. The increased intestine weight at 42 d may reflect a physiological adaptation of the SI to the bioactive phytonutrients in CPE, potentially reflecting enhanced tissue development or metabolic activity.

To further examine a possible adaptive intestinal response to dietary treatment, we evaluated intestinal histomorphology of SI and cecum as a complementary analysis to the observed variations in intestinal weight. The SI is the primary site of nutrient digestion and absorption, and the cecum contributes to water absorption and supports microbial fermentation. Standard indicators of intestinal development include VH, VW, and CD, where taller, wider villi and shallower crypts generally indicate improved absorptive capacity and epithelial health (Gao et al., 2008; Zeitz et al., 2015).

**Fig. 1.**
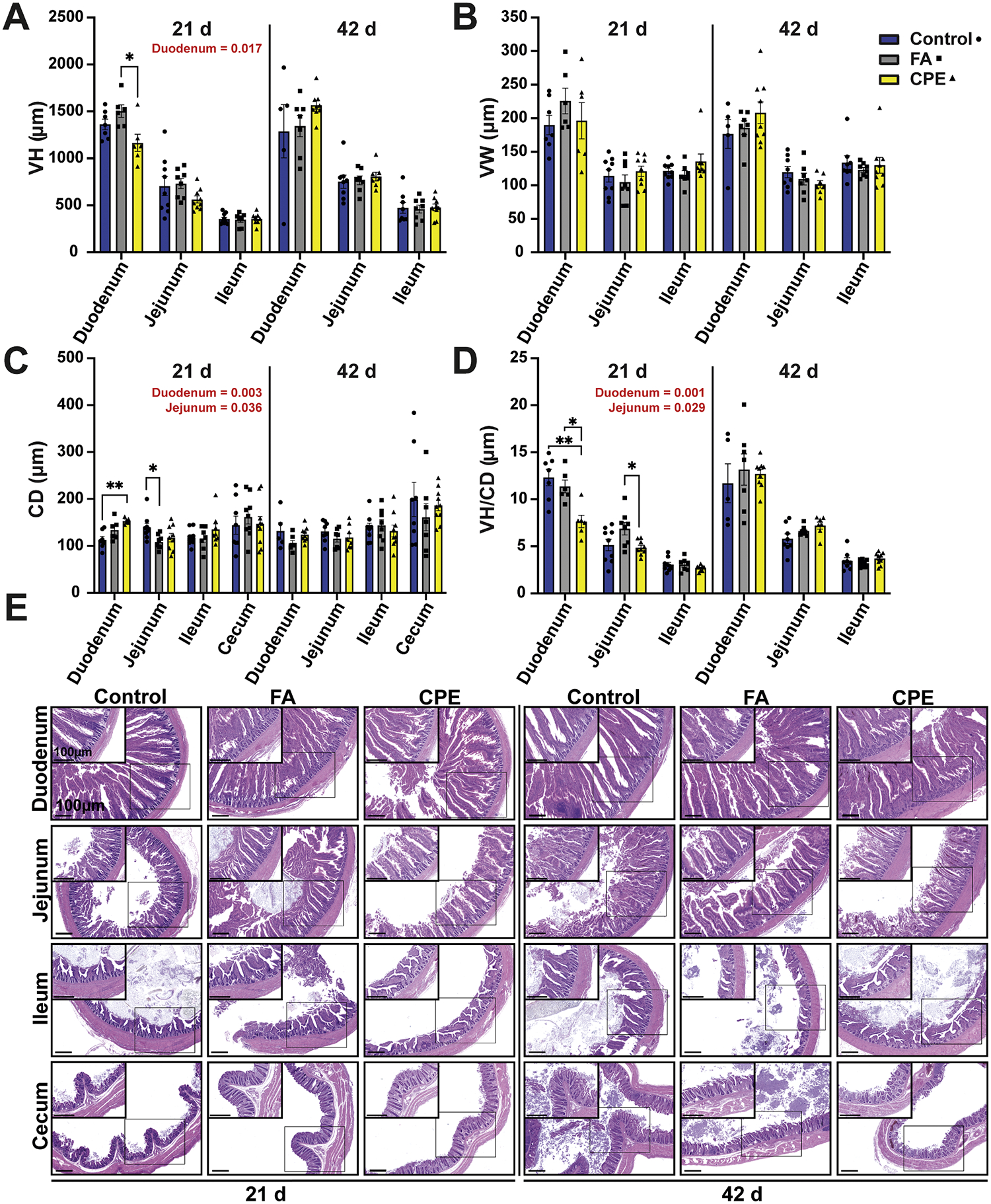
Small intestinal and cecal morphometry in broilers supplemented with pure FA or FA-rich CPE at 21 and 42 d. Measurements (μm) of VH (A), VW (B), CD (C), and VH/CD ratio (D) were obtained from duodenal, jejunal, ileal, and cecal sections. Representative hematoxylin and eosin-stained histological images of each intestinal section at 100x and 200x magnification (bump-outs) are shown for 21 and 42 d (E). Bars represent mean values with error bars indicating SEM. Red values indicate P-value from one-way ANOVA; asterisks indicate differences among dietary treatments (*P < 0.05; **P < 0.01).

Our results showed that pure FA and CPE supplementation affected intestinal morphology in an age- and region-specific manner d (Fig. 1A–D, with representative images shown in Fig. 1E). At 21 d, main effects of treatment were observed for duodenal VH (*P* = 0.017), CD (*P* = 0.003), and VH/CD (*P* = 0.001), as well as jejunal CD (*P* = 0.036) and VH/CD (*P* = 0.029) (Fig. 1A, C, and D). In particular, duodenal VH in CPE-fed birds were shorter compared with the FA group (*P* < 0.050; Fig. 1A), with deeper CD relative to the Control group (*P* < 0.010; Fig. 1C). As a result, the VH/CD ratio was lower in CPE-fed birds relative to both Control and FA-fed birds (*P* < 0.050; Fig. 1D). These morphological changes may suggest increased epithelial cell turnover and transient intestinal remodeling in response to early exposure to bioactive compounds present in CPE, which may coincide with dynamic post-hatch intestinal adaptation reported to occur in broiler chickens (Gaweł et al., 2025). In contrast, FA supplementation was associated with shallower CD when compared with Control and a higher jejunal VH/CD ratio at 21 d relative to CPE-fed birds (*P* < 0.050; Fig. 1C, D), possibly indicating a more stable intestinal architecture that may support efficient nutrient absorption during early development.

The observed changes in intestinal histomorphology are consistent with the reduced BWG observed at 7 d in CPE-supplemented birds. In broilers, a higher VH/CD ratio is generally associated with improved nutrient absorption efficiency and intestinal health, reflecting greater absorptive surface area and reduced epithelial turnover, as suggested by others (Oliveira et al., 2008; Silva et al., 2009; Prakatur et al., 2019). Therefore, the reduced VH/CD ratio in CPE-supplemented birds likely reflects a temporary reduction in absorptive efficiency during early post-hatch development, which may have contributed to the reduced BWG observed during the first 7 d post-hatch (Table 2).

Conversely, the improvement in jejunal VH/CD ratio observed with pure FA supplementation relative to the CPE-fed group may have contributed to increased BWG observed at later ages, particularly at 35 d and during the 22–42 d interval (Table 2). A previous study in mice showed that FA enhances antioxidant capacity by activating the nuclear factor-erythroid 2-related factor 2/Hemeoxygenase-1 signaling pathway, thereby reducing oxidative stress through ROS elimination (He et al., 2018). Furthermore, FA has been reported to promote digestive function by enhancing digestive enzyme activity, improving digestion and absorption of substrates critical for supporting growth and development in grouper fish (Fu et al., 2022). While our study did not directly assess the antioxidant and digestive enzyme–related pathways described in previous reports, these mechanisms may partially explain the improved performance observed with dietary FA supplementation beyond the first wk post-hatch. Notably, the relevance of these mechanisms to intestinal histomorphology remains unclear, as research directly investigating the impacts of FA supplementation on intestinal histomorphology in broiler chickens appears to be lacking, which warrants further investigation.

### Circulating lipid profiles of broilers fed diets containing pure FA or FA-rich CPE

Circulating lipid profiles were measured to evaluate the metabolic response to dietary supplementation with pure FA or FA-rich CPE relative to the Control diet. No significant differences in TC, TG, or NEFA were observed among treatment groups at 21 and 42 d (*P* > 0.050, Table 4). At 21 d, NEFA tended to be lower in CPE-fed birds (*P* = 0.094); whereas at 42 d, NEFA values were numerically lower in CPE-fed birds but not significant (*P* = 0.125). Both FA and CPE groups showed numerically higher TC values at 42 d (*P* = 0.056).

NEFA is a critical indicator of lipid metabolism in poultry, serving as an important energy substrate, where levels in circulation provide insights into overall metabolic status and energy balance (Langslow et al., 1970; Krasnodȩbska-Depta and Koncicki, 2000; Sutherland et al., 2019). The trend toward reduced NEFA levels in CPE-supplemented birds may indicate a more stable energy status, particularly at 42 d when these birds also showed numerically higher FI relative to the FA group (Table 2). It is possible that CPE-fed birds were in a positive energy balance and therefore did not require NEFA mobilization. However, this should be interpreted with caution, as the birds were not fasted prior to blood collection, which may have influenced NEFA levels (Serr et al., 2009; Torchon et al., 2016). Further, since FI was similar across groups at 21 d, energy balance is unlikely to explain the trend toward lower NEFA levels observed at this earlier time point (*P* > 0.050). Nonetheless, the consistent trend toward reduced NEFA at both time points may reflect early and sustained metabolic response to CPE, rather than a delayed, time-dependent effect.

Meanwhile, the tendency toward higher TC levels at 42 d in both FA and CPE groups may reflect mild upregulation of lipid synthesis or transport pathways associated with phenolic compound metabolism. Earlier studies demonstrated that phytochemical-rich plant extracts reduced TC in serum of chickens through regulating the expression of genes related to fat synthesis and decomposition (Li et al., 2013; Hassan et al., 2018; Ding et al., 2023). Given that the active components and the extraction method of plant polyphenols vary widely, which in turn could affect their biological efficacy, further research is needed to clarify the role of FA-rich CPE in regulating lipid metabolism in broilers. It is also possible that the bioactive components of CPE require time to accumulate in tissues before exerting systemic metabolic effects, which warrants investigation across varying inclusion levels and timing of exposure.

### Effects of dietary pure FA and FA-rich CPE on hepatic cytokine and redox gene expression

**Fig. 2.**
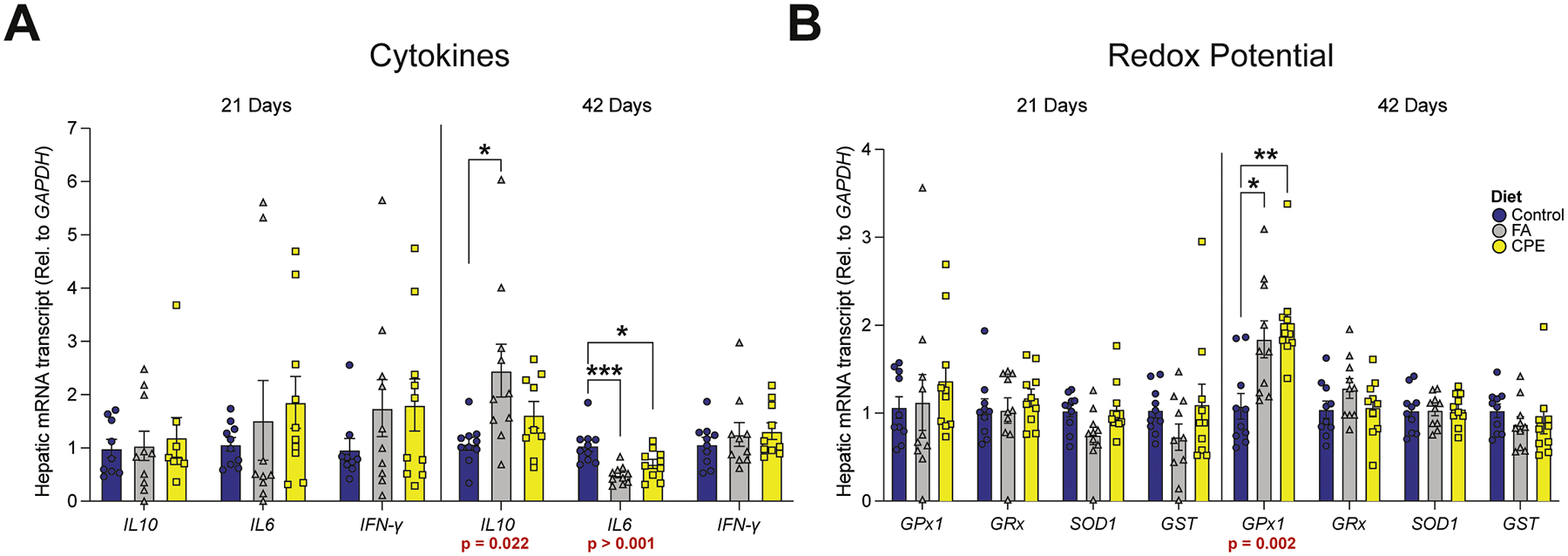
Hepatic mRNA transcript levels of inflammatory cytokines and redox enzymes in broilers fed pure FA or FA-rich CPE at 21 and 42 d. Transcript levels of cytokine genes *IL6* (Interleukin-6), *IL10* (Interleukin-10), and *IFNγ* (Interferon-γ) relative to the housekeeping gene *GAPDH* (A). Transcript levels of redox enzyme genes *GPX1* (glutathione peroxidase1), *GR* (glutathione reductase), *SOD1* (superoxide dismutase1), and *GST* (glutathione transferase) relative to the housekeeping gene *GAPDH* (B). Data are expressed as ΔΔCT relative to Control. Values represent means ± SEM, *n* = 10 birds per treatment and age. Red values indicate P-value from one-way ANOVA; asterisks indicate differences among dietary treatments (*P < 0.05; **P < 0.01; ***P < 0.001).

Given the observed numerical trends in circulating lipid parameters, we further examined hepatic gene expression related to cytokine signaling and redox status to better understand the downstream biological influence of FA and CPE supplementation. At 21 d, no significant differences were detected in hepatic cytokine and redox potential gene expression between groups (Fig. 2A, B). However, at 42 d, FA-fed birds showed significant upregulation of interleukin-10 (***IL10***) compared with the Control group, while both FA- and CPE-supplemented birds exhibited significant downregulation of interleukin-6 (***IL6***) expression relative to the Control group (Fig. 2A). Further, both FA and CPE supplementation significantly upregulated hepatic gene expression of glutathione peroxidase 1 (***GPX1***) relative to Control diet at 42 d (Fig. 2B). These changes indicate that both FA and CPE may contribute to hepatic immune regulation and antioxidant responses in a time-dependent manner.

Immunological stress, indicated by activation of pro- and anti-inflammatory cytokines, is a well-known factor that can impact animal performance. Pro-inflammatory cytokines, such as IL-6, are typically generated by classically activated M1 macrophages, while IL-10 is commonly associated with the anti-inflammatory potential of alternatively activated M2 macrophages (Yunna et al., 2020). Consistent with previous findings in poultry (Yi et al., 2025), the observed downregulation of hepatic *IL6* and upregulation of *IL10* mRNA transcript levels in response to dietary CPE and FA supplementation at 42 d suggest a potential attenuation of pro-inflammatory signaling. This may suggest supplemental phenolic compounds, particularly FA, elicit immunomodulatory effects later in life because these compounds require time to accumulate in tissues before exerting systemic metabolic and immune responses. However, previous studies have reported inconsistent effects of FA on cytokine responses, which could be related to species-specific responses and dietary inclusion levels. For example, Du et al. (2022) found that dietary inclusion of 80 mg/kg FA drove elevated levels of pro-inflammatory cytokines TNF-α and IL-2 in the circulation of broilers. In contrast, another study showed that ducks exposed to 200, 400, and 800 mg/kg of dietary FA had decreased circulating levels of IL-6 and IL-2 in a dose-dependent manner (Liu et al., 2021b). Additional studies are needed to parse out the dose- and species-specific immunomodulatory responses to FA and other related phenolic compounds.

Oxidative stress is primarily caused by excessive ROS accumulation that exceeds the scavenging capacity of the antioxidant defense system, disrupting redox homeostasis (Nisar et al., 2013; Surai et al., 2019). This antioxidant defense system includes both enzymatic and non-enzymatic components. Here, we show that both CPE and FA supplementation upregulated hepatic expression of *GPX1*, a key intracellular antioxidant enzyme that reduces hydrogen peroxide (Huang et al., 2018) and lipid peroxides (Lubos et al., 2011), suggestive of enhanced antioxidant capacity later in life. Similar findings have been reported in other species with pure FA supplementation. For instance, Liu et al. (2021b) observed anti-inflammatory effects of FA supplementation in ducks. These birds also showed increased serum glutathione and GPx1 activity. Further, oral supplementation with 25 or 50 mg/kg FA enhanced hepatic superoxide dismutase (**SOD**), catalase (**CAT**), and GPx1 activities in rats under oxidative challenge with methotrexate (Mahmoud et al., 2020). Similarly, dietary supplementation with FA increased serum and hepatic activities of SOD, CAT, and GPx1 in weaning piglets (Wang et al., 2020). However, in our study, hepatic expression of other redox potential genes did not differ between treatment groups, suggesting that the antioxidant effects of CPE and FA may be limited to specific pathways or require different inclusion rates and conditions for broader activation.

### Fecal microbiota diversity in response to pure FA or FA-rich CPE dietary supplementation

To explore potential downstream effects of pure FA and FA-rich CPE supplementation, we evaluated fecal and cecal microbial communities across different growth phases. Microbial richness, evenness, and composition were analyzed to determine whether supplementation with phenolic compounds influenced gut microbiota development and potential links to growth performance.

**Fig. 3.**
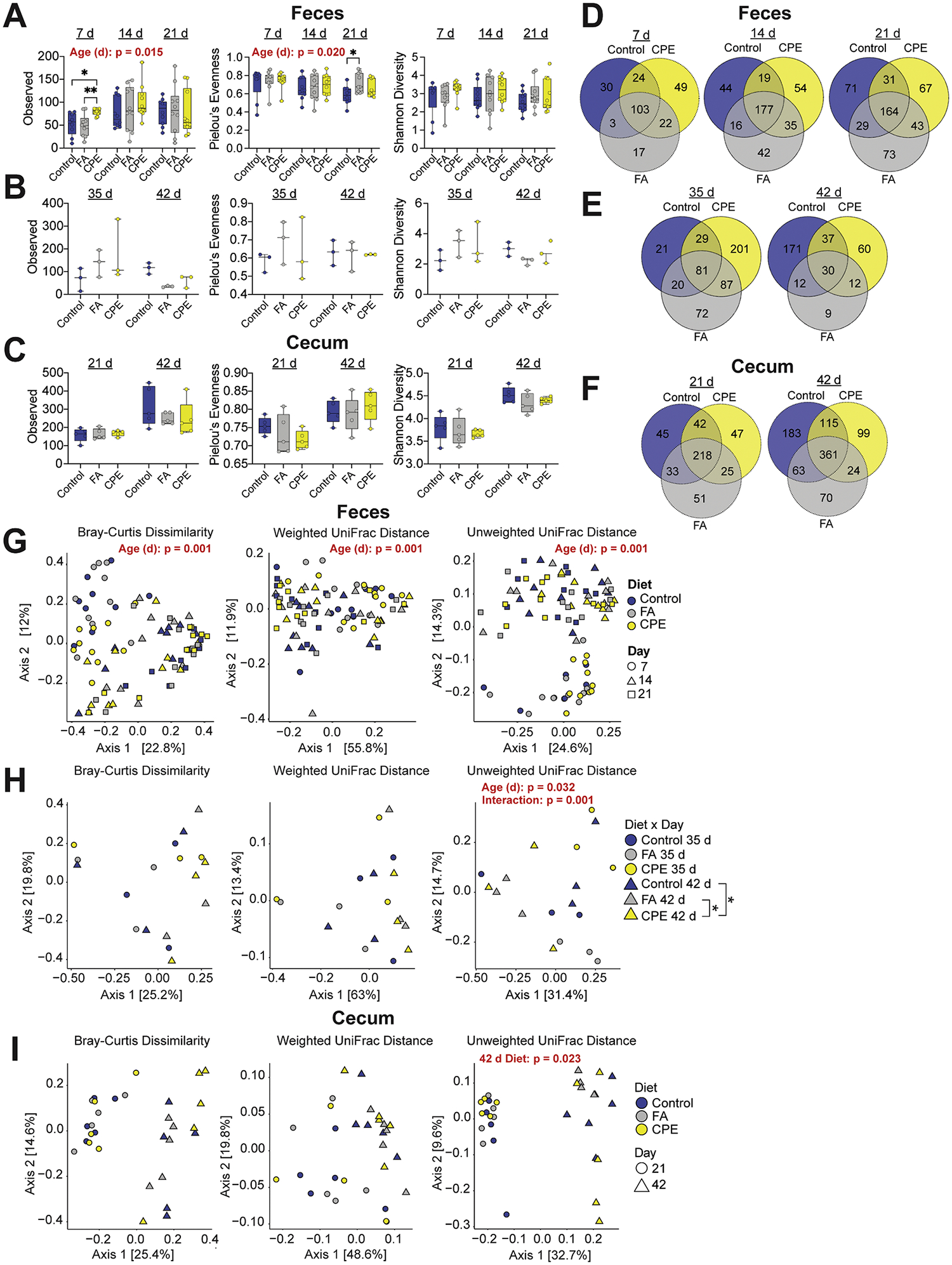
16S rRNA gene amplicon sequencing analysis of microbial diversity in feces and cecal contents from broilers fed pure FA or FA-rich CPE. Alpha diversity metrics (Observed species, Pielou’s evenness, and Shannon diversity index) across treatments and age are shown for cage/pen-level fecal samples collected from 7–21 d (A) and 35–42 d (B), and for bird-level cecal contents at 21 and 42 d (C). Venn diagrams illustrate the number of shared and unique ASVs across treatment groups in cage/pen-level fecal samples from 7–21 d (D) and 35–42 d (E), and in cecal contents at 21 and 42 d (F). Beta-diversity principal coordinate analysis (PCoA) based on Bray-Curtis dissimilarity and unweighted and weighted UniFrac distances is shown for fecal samples collected from 7–21 d (G) and 35–42 d (H), and for cecal contents collected at 21 and 42 d (I). Box-and-whisker plots represent means and interquartile ranges, with individual points representing biological replicates. Red values indicate P-values from two-way ANOVA; asterisks indicate differences among dietary treatments (*P < 0.05; **P < 0.01).

Alpha-diversity in feces was primarily influenced by bird age (d), with significant main effects observed in Observed Species (*P* = 0.050) and Pielou’s Evenness (*P* = 0.005) from 0 to 21 d (Fig. 3A). Supplementation with CPE transiently increased unique ASVs at 7 d relative to both Control and FA groups (*P* < 0.010 and *P* < 0.050, respectively; Fig. 3A, left panel). By 21 d, FA supplementation increased evenness relative to the Control (*P* < 0.050; Fig. 3A, middle panel). No treatment differences were detected at 35 and 42 d (Fig. 3B). In cecal contents, an upward trend in alpha-diversity metrics was observed from 21 d to 42 d, but diet effects were not observed (Fig. 3C).

At 7 d, 103 ASVs were shared across all dietary groups, with CPE-supplemented birds exhibiting the greatest number of unique ASVs (49) compared to Control (30) and FA (17) (Fig. 3D). Similar trends were observed at 14 d, while numbers of unique ASVs were comparable across all groups at 21 d (Fig. 3D). At 35 and 42 d, 81 and 30 ASVs were shared across groups, respectively, with CPE driving the most unique ASVs at 35 d, which was replaced by Control at 42 d (Fig. 3E). In cecal contents, 218 and 361 ASVs were shared across diet groups at 35 and 42 d, respectively, with patterns of unique ASVs generally mirroring trends observed in feces (Fig. 3F).

Beta-diversity analysis from 0–21 d showed a significant main effect of age for both non-phylogenetic (Bray-Curtis Dissimilarity) and phylogenetic (Unweighted and Weighted UniFrac) distance metrics (*P* < 0.001; Fig. 3G). At 42 d, a significant age x diet interaction (*P* = 0.001) was detected for Unweighted UniFrac, with CPE-fed birds differing from both Control and FA-fed counterparts (*P* < 0.050; Fig. 3H). In cecal contents, dietary treatment affected Unweighted UniFrac distances at 42 d (*P* = 0.023), though pairwise differences between groups were not significant (Fig. 3I).

These findings confirm that microbial richness and evenness, indicators of community complexity, are primarily influenced by bird age in both feces and cecal contents (Zhou et al., 2021; Li et al., 2022). However, early life supplementation with phenolic compounds may alter these dynamics, as observed at 7 d. Our results align with a previous study showing that early interventions, such as exposure to antibiotics, polyphenol-rich essential oils, or a *Bacillus subtilis* probiotic, can alter beta-diversity over time in feces without affecting Shannon diversity at 7 d, with no interaction between treatment or bird age (Fonseca et al., 2024). Conversely, tannic acid supplementation has been reported to alter ileal Shannon diversity at 35 d in broilers (Xu et al., 2025). While we did not assess ileal microbiota, polyphenol-induced shifts may occur in the proximal intestine, with effects potentially increasing with age as gastrointestinal transit slows in broilers (Vergara et al., 1989), and these effects may not be reflected in fecal or cecal microbiota composition.

### Effects of pure FA or FA-rich CPE dietary supplementation on fecal microbiota taxonomic composition at the phylum and family levels

**Fig. 4.**
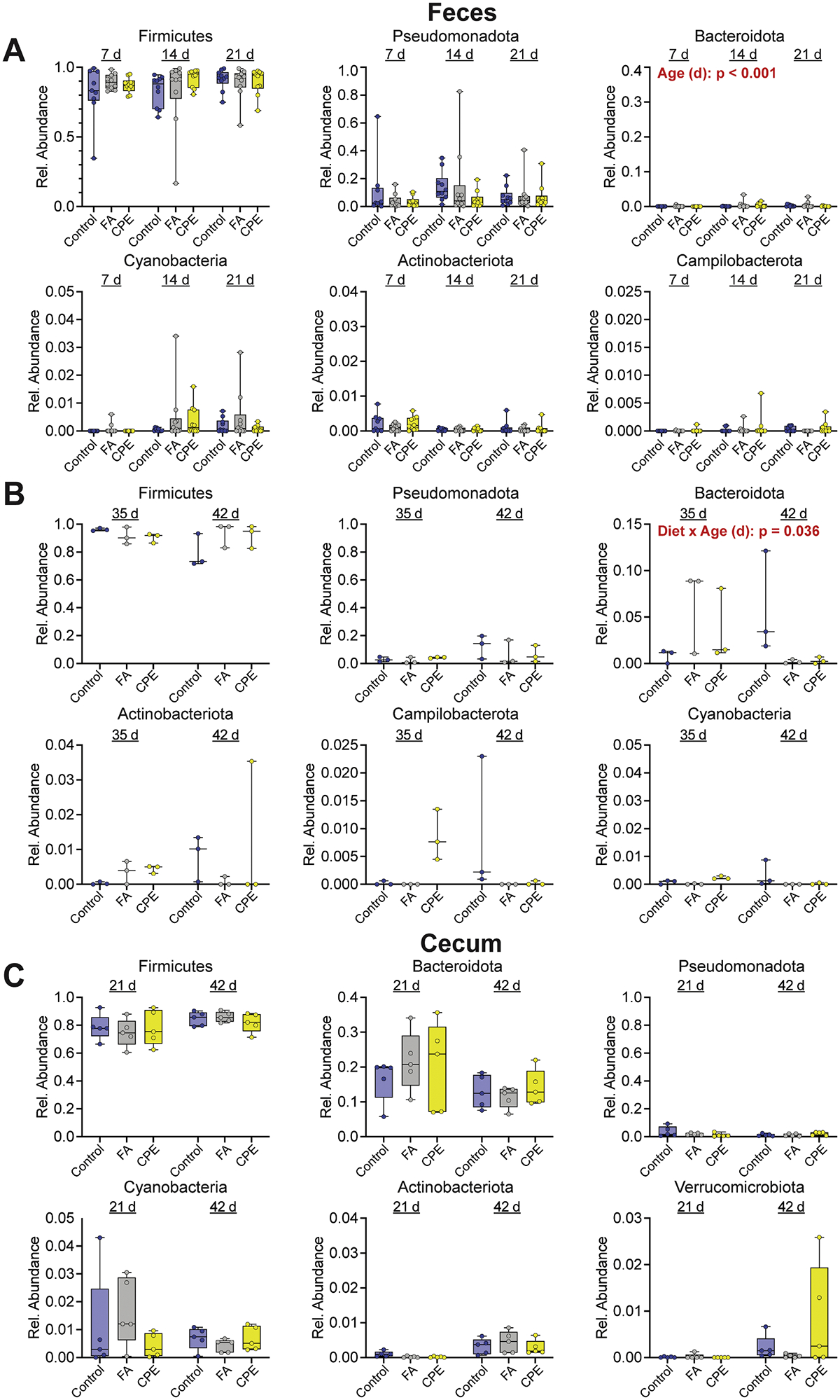
Bacterial phylum-level composition in feces and cecal contents of broilers fed pure FA or FA-rich CPE. Relative abundance of the top 6 bacterial phyla across treatment groups and age in cage/pen-level fecal samples from 7–21 d (A) and 35–42 d (B), and in cecal contents at 21 and 42 d (C). Box and whisker plots represent means and interquartile range; individual data points represent biological replicates. Red values indicate P-values from two-way ANOVA; asterisks indicate differences among dietary treatments (*P < 0.05; **P < 0.01).

At the phylum level, fecal Bacteroidota abundance exhibited an age-dependent decrease in feces regardless of diet, with a decrease first observed at 14 d (*P* < 0.001; Fig. 4A, top six phyla shown for all ages). A significant age x diet interaction from 35 to 42 d was also evident for Bacteroidota abundance in feces (*P* = 0.036) and although pairwise comparisons were not statistically significant, a numerical decline in FA- and CPE-supplemented groups was observed relative to Control (Fig. 4B). Cecal phylum level composition was largely unaffected by diet or age (Fig. 4C). Collectively, these findings indicate that phylum-level shifts in fecal microbial composition are predominantly driven by broiler age, with limited influence of dietary supplementation, consistent with Ranjitkar et al. (2016).

**Fig. 5.**
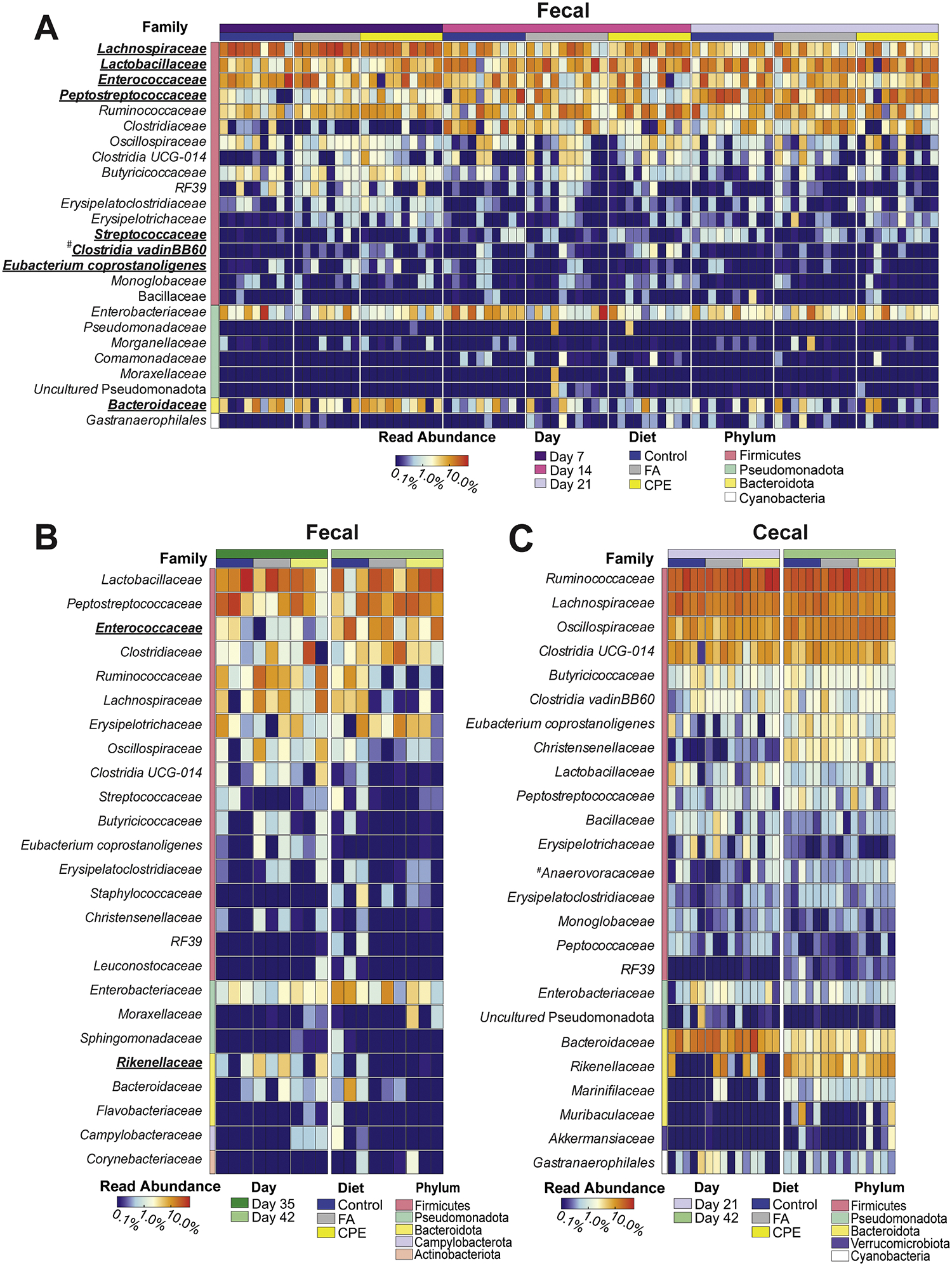
Bacterial family-level composition in feces and cecal contents of broilers fed pure FA or FA-rich CPE. Heatmaps represent normalized relative abundances of the top 25 bacterial families across treatment groups and age in cage/pen-level fecal samples from 7 to 21 d (A) and 35–42 d (B), and in cecal contents from individual birds at 21 and 42 d (C). Bold and underlined taxa represent a significant main effect of age and # represents a significant main effect of diet (*P* < 0.05).

Family-level analysis from 7 to 21 d revealed age-related effects on the relative abundance of several Firmicutes families, including *Lachnospiraceae, Lactobacillaceae, Enterococcaceae, Peptostreptococcaceae, Streptococcaceae, Clostridia vandin BB60*, and *Eubacterium coprostanoligenes*, as well as *Bacteroidaceae*, a member of the Bacteroidota phylum (*P* < 0.050; Fig. 5A). A significant main effect of diet was also detected for *Clostridia vandin BB60* (*P* < 0.050, Fig, 5A). From 35 to 42 d, age remained a significant driver of microbial composition, particularly for *Rikenellaceae* and *Enterococcaceae* (*P* < 0.050; Fig, 5B). In cecal contents, a diet effect was observed for *Anaerovoracaceae*, with increased abundance in CPE-fed birds (*P* < 0.050, Fig, 5C).

Overall, our data show bacterial family-level composition is largely driven by developmental age, with early-life reductions in *Bacteroidaceae* and age- or diet-associated shifts in Firmicutes families that did not overtly shift phylum-level composition. Longitudinal studies have shown that gut microbial community composition in broilers changes markedly with age, where richness and community structure evolve throughout the production cycle, and that age often exerts a stronger influence on overall microbiota patterns than diet alone (Awad et al., 2016; Shang et al., 2018; Zhou et al., 2021), which reinforces the current finding. While others have reported stable *Bacteroidaceae* abundance during early broiler development (Fonseca et al., 2024), such discrepancies may reflect differences in broiler genetic background, housing conditions, or dietary composition, highlighting the need for further investigation into the environmental and host factors that shape microbial colonization dynamics. Despite regional variation along the poultry GIT, fecal and cecal samples shared several dominant families linked with gut health regardless of diet, including *Lachnospiraceae* and *Ruminococcaceae*, which are known short-chain fatty acid (**SCFA**) producers (Diaz Carrasco et al., 2019; Rychlik, 2020). Consistent with previous reports associating these families with improved FCR (Stanley et al., 2016), they remained abundant across ages and dietary treatments in the present study (Fig. 5), supporting the conclusion that age-related microbial maturation predominates over dietary effects.

### Dietary FA and CPE select for specific gut microbes and enrich phenolic-degrading taxa early in life

**Fig. 6.**
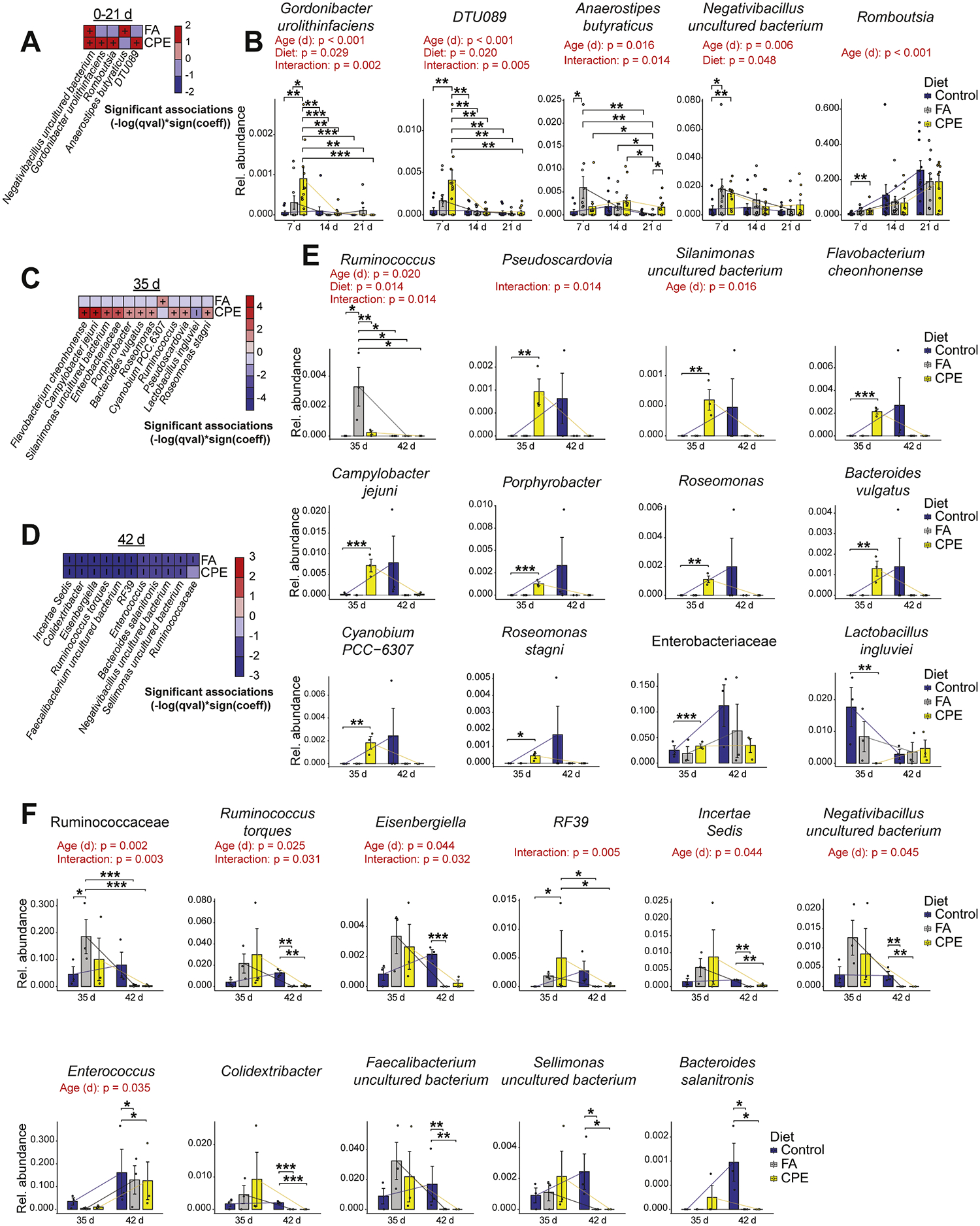
Age and diet-associated differences in fecal bacterial taxa in broilers fed pure FA or CPE. Heatmaps show bacterial taxa identified as differentially abundant relative to the Control treatment in cage/pen-level feces collected at 7 d (A), 35 d (C), and 42 d (D) using MaAsLin2. Relative abundance of differentially abundant taxa across dietary treatments and age is shown for cage/pen-level fecal samples collected at 7–21 d (B) and 35–42 d (E–F). Plus (+) and minus (−) symbols in heatmaps in panels A and C-D indicate significant positive or negative associations, respectively, relative to the Control (*P* < 0.05). Bar plots represent means ± SEM, with individual points representing biological replicates. Red values indicate P-values from two-way ANOVA; asterisks denote significant differences among dietary treatments (*P < 0.05, **P < 0.01, **P < 0.001). In panels B, E, and F, when only significant main effects of age (d) or diet were detected, MaAslin2 pairwise comparisons within age (d) are shown.

Analysis using MaAsLin2 identified differentially enriched microbial taxa in response to FA and CPE supplementation across ages, indicating an early-life microbial response to polyphenol supplementation. At 7 d, CPE supplementation enriched *Gordonibacter urolithinfaciens, Romboutsia*, and DTU089, while both CPE- and FA-fed birds showed increased *Negativibacillus* relative to Controls (Fig. 6A). In contrast, *Anaerostipes butyraticus* was selectively enriched in FA-fed birds (Fig. 6A).

Both *G. urolithinfaciens* and *DTU089* exhibited significant age x diet interactions (*P* = 0.002 and *P* = 0.005, respectively), characterized by peak abundance at 7 d in CPE-fed birds followed by declines by 21 d (*P* < 0.050, Fig. 6B). *A. butyraticus* also showed an age x diet interaction (*P* = 0.014), with enrichment in FA-fed birds relative to Control at 7 d (*P* < 0.010), followed by declining abundance from 7 to 21 d, while CPE supplementation resulted in modest enrichment at 21 d compared with FA-fed birds (*P* < 0.05, Fig. 6B). *Negativibacillus* (uncultured bacterium) abundance was influenced both by age (*P* = 0.048) and diet (*P* = 0.005), with increases in both FA- and CPE-fed birds relative to Control at 7 d (*P* < 0.050 and *P* < 0.010, respectively), whereas *Romboutsia* was primarily affected by age (*P* < 0.001) (Fig. 6B).

Notably, enrichment of *G. urolithinfaciens* at 7 d (Fig. 6B) coincided with improved early BWG observed in CPE-fed birds relative to those receiving pure FA at 7 d (Table 2). A previous study showed that consumption of polyphenol-rich foods, such as walnuts, promotes the expansion of *G. urolithinfaciens* in humans (Liu et al., 2024). This bacterium has also been shown in humans to directly catabolize dietary ellagitannins into bioactive urolithins, enhancing polyphenol bioavailability and absorption (García-Villalba et al., 2020), suggesting a likely mechanism by which *G. urolithinfaciens* may interact with phenolic substrates in avian hosts.

The early co-enrichment of *DTU089* with *G. urolithinfaciens*, despite a lack of direct evidence for polyphenol metabolism, suggests a potential supporting role in polyphenol transformations within the gut (Calvete-Torre et al., 2022). *A. butyraticus*, a known butyrate producer, has been linked to improved intestinal health in broilers via SCFA production (Eeckhaut et al., 2010; De Maesschalck et al., 2015). *Negativibacillus*, a member of the Oscillospiraceae family, has been associated with fiber fermentation (Liu et al., 2021a; Lysko et al., 2022) and probiotic supplementation in poultry (Zhu et al., 2020). However, its relationship with performance appears context-dependent, with reports linking its abundance to both altered BW responses (Farkas et al., 2022; Yang et al., 2023; Deryabin et al., 2024) and subclinical enteritis (Wang et al., 2021; Zhao et al., 2022). *Romboutsia*, a member of the Peptostreptococcaceae family, has been associated with improved growth performance and gut health in broilers (Deng et al., 2023; Song et al., 2024). Zhang et al. (2021) showed that *Bacillus subtilis* supplementation increased both growth performance and *Romboutsia* abundance, suggesting a link to enhanced fermentation capacity.

At later ages, MaAsLin2 analysis revealed broader, but largely transient diet-associated shifts in fecal microbiota composition (Fig. 6C, D). At 35 d, CPE supplementation enriched multiple taxa spanning the Pseudomonadota and Bacteroidota phyla and reduced *Lactobacillus ingluviei*, a Firmicutes member associated with improved digestion and nutrient metabolism in poultry (Murakami et al., 2024) (Fig. 6C). Supplementation with FA enriched a single *Ruminococcus* ASV (Fig. 6C). By 42 d, both FA and CPE supplementation were associated with reduced abundance of several Firmicutes taxa, including *Ruminococcus torques, Faecalibacterium* (uncultured bacterium)*, Enterococcus*, and related genera, as well as *Bacteroides salanitronis*, with only FA-fed birds exhibiting a significant reduction in *Ruminococcaceae* relative to Controls (Fig. 6D). No significant dietary effects were detected in cecal contents at either age (data not shown).

Comparisons of 35 d taxonomic indicators showed that *Ruminococcus* abundance was significantly impacted by age x diet interaction (*P* = 0.014), with enrichment in FA-fed birds at 35 d followed by a convergence across treatments at 42 d (Fig. 6E). An age x diet interaction was also observed for *Pseudoscardovia* (*P* = 0.014), whereas *Silanimonas* (uncultured bacterium) exhibited a main effect of age (*P* = 0.016), with higher abundances in CPE-fed birds at 35 d relative to Control (*P* < 0.010) (Fig. 6E). Consistent trends were also noted for several additional CPE-enriched taxa at 35 d, including *C. jejuni* (*P* < 0.001), alongside members of the Pseudomonadota and Bacteroidota phyla, such as *Porphyrobacter, Bacteroides vulgatus, Cyanobium PCC.6307, Roseomonas*, and *Enterobacteriaceae* (*P* < 0.010) (Fig. 6E). In contrast, *L. ingluviei* abundance was significantly reduced in CPE-fed birds at 35 d compared to Controls (*P* < 0.001), with abundances converging across treatments by 42 d (Fig. 6E).

Distinct taxonomic indicators were identified at 42 d, with no overlap with those observed at 35 d (Fig. 6F). Several Firmicutes taxa, including *Ruminococcaceae, R. torques*, and *Eisenbergiella* exhibited significant age x diet interactions (*P* = 0.003, *P* = 0.031, and *P* = 0.032, respectively). *Ruminococcaceae* was enriched by FA supplementation at 35 d compared to Controls (*P* < 0.050), followed by a marked reduction in both FA- and CPE-fed birds at 42 d (*P* < 0.001; Fig. 6F). By contrast, *R. torques* was significantly enriched in Control-fed birds relative to both FA- and CPE-fed birds (*P* < 0.010), whereas *Eisenbergiella* abundance was elevated in Control-fed birds compared to FA-fed birds only (*P* < 0.001) (Fig. 6F). An age x diet interaction was also observed for the Tenericutes taxon *RF39* (*P* = 0.005), with enrichment in CPE-fed birds at 35 d relative to the Control (*P* < 0.050), followed by reduced abundance in both FA- and CPE-fed birds at 42 d (*P* < 0.050, Fig. 6F). Significant main effects of age were observed for *Negativibacillus* (uncultured bacterium), *Incertae Sedis*, and *Enterococcus* at 42 d (*P* < 0.050), with lower abundances in FA- and CPE-fed relative to Controls (Fig. 6F). Similar, though non-significant, trends were observed for *Colidextribacter, Faecalibacterium* (uncultured bacterium)*, Sellimonas* (uncultured bacterium), and *Bacteroides salanitronis*.

At 35 d, *C. jejuni*, a foodborne pathogen that colonizes the avian gut asymptomatically (Evans and Sayers, 2000; Al Hakeem et al., 2024; Awad et al., 2025), was enriched by CPE (Fig. 6C, E), which was unexpected given that dietary polyphenols generally suppress Campylobacter colonization (Klančnik et al., 2012; Navarro et al., 2015; Oh and Jeon, 2015). Interestingly, *C. jejuni* expansion and reduced *L. ingluviei* in CPE-fed birds coincided with duodenal histological changes at 21 d, including reduced VH, increased CD, and decreased VH/CD ratio (Fig. 1), which preceded reduced growth performance at 35 d (Table 2). By 42 d, both FA and CPE supplementation were associated with reductions in several Firmicutes taxa commonly linked to fermentation and SCFA production, including Ruminococcaceae*, R. torques*, and *F. prausnitzii* (Fig. 6F) (Torok et al., 2011; Liu et al., 2021c; Montso et al., 2022), suggesting that prolonged phenolic exposure may influence microbial fermentative capacity during the finisher phase. Further research is needed to determine the functional implications of these microbial changes for intestinal health.

Collectively, these results indicate that fecal microbial composition is primarily shaped by developmental age, with secondary and largely transient modulation by phenolic supplementation, consistent with previous reports in broilers (Ranjitkar et al., 2016; Rychlik, 2020). Although bird age appeared to significantly influence microbial community membership, the housing transition to floor pens at 22 d and the use of a mash diet rather than a more standard pelletized diet may have confounded some of the observed treatment effects. Further investigation is necessary to disentangle the age-related and tissue-specific effects of early vs. prolonged polyphenol exposure on gut microbial ecology and systemic metabolic responses in broilers.

## Conclusions and applications

1. Supplementation with FA-rich CPE enhances early growth performance in broilers compared to pure FA, with minimal effects relative to the Control. Extended CPE feeding, however, may impair growth performance relative to pure FA, potentially via altered intestinal development and nutrient absorption.
2. Neither CPE nor FA impacted plasma lipid profiles, but prolonged exposure to either supplement elicited positive immunomodulatory and antioxidant effects, including decreased IL6 expression and upregulation of GPx1 expression.
3. Bird age primarily drove changes in microbial diversity; however, CPE transiently enriched unique community members early in life, including phenolic-degrading taxa *G. urolithinfaciens* and *Romboutsia*.
4. Both FA and CPE reduced the abundance of fermentative Firmicutes in feces later in life, while CPE promoted expansion of *C. jejuni*, a known foodborne pathogen.
5. FA-rich CPE influenced early-life growth performance and microbial composition, highlighting its potential as a non-antibiotic feed additive. However, these findings were obtained under controlled research conditions with replication levels standard for poultry feeding trials and should be considered exploratory. Further studies with larger sample sizes are needed to validate effects under commercial conditions and determine optimal dosage and timing for maximizing CPE functional benefits in broilers.

## Supplementary Material

1

Supplementary material associated with this article can be found in the online version at doi:10.1016/j.japr.2026.100683.

## Figures and Tables

**Table 1:** Ingredients and composition (%) of basal diet*.

Ingredients	0–10 d	11–24 d	25–42 d
Yellow corn	54.4	57.33	60.08
Soybean meal, 47%	30.00	22.30	23.00
Corn gluten, 60%	6.63	9.38	6.55
Corn oil	2.42	2.85	4.48
Wheat middling	2.00	3.90	2.00
limestone	1.50	1.37	1.33
Monocalcium phosphate	1.80	1.65	1.45
Common salt	0.20	0.22	0.20
Sodium Bicarbonate	0.20	0.20	0.20
Premix**	0.10	0.10	0.10
DL- Methionine, 98%	0.21	0.10	0.13
Lysine, HCl, 78%	0.34	0.36	0.28
Choline chloride	0.24	0.24	0.24
Chemical composition			
ME, Kcal/Kg	3000.50	3100.57	3200.06
CP%	23.00	21.50	20.00
EE%	4.95	5.56	7.17
CF%	3.52	3.22	3.15
Ca%	0.95	0.86	0.81
Available P%	0.48	0.44	0.40
Lysine%	1.28	1.14	1.06
Methionine%	0.56	0.47	0.45

* Basal diet was supplemented with either 100 mg/kg FA or 600 mg/kg Corn Pericarp Extract, standardized to provide 100 mg/kg FA.

** Vitamin and mineral premix per kg of diet: vitamin A, 12,000 IU; vitamin D3, 5000 IU; vitamin E, 80 IU; vitamin K3, 3.20 mg; thiamine, 3.20 mg; riboflavin, 8.60 mg; pantothenic acid, 20 mg; folic acid, 2.20 mg; pyridoxine, 4.3 mg; niacin, 65 mg; vitamin B12, 0.017 mg; biotin, 0.22 mg; choline, 1650 mg; Fe, 20 mg; Cu, 16 mg; Mn, 120 mg; Zn, 110 mg; I, 1.25 mg; Se, 0.30 mg. ME: Metabolizable energy; CP: Crude protein; EE: Ether extract; CF: Crude fiber; Ca: Calcium; P: Phosphorus.

**Table 2:** Effect of dietary pure FA and CPE on broiler growth performance.

Parameter	Age, d	Control	FA 100 mg/kg	CPE 600 mg/kg	Pooled SEM	*P*-value
BW, g/bird	Initial	43.46	43.37	43.16	0.103	0.830
	7	180.35^a^	169.17^b^	182.85^a^	1.700	0.002
	14	458.04	458.10	456.21	4.617	0.873
	21	907.39	901.73	900.20	8.742	0.926
	28	1481.33	1527.00	1451.67	34.900	0.312
	35	2257.67^ab^	2346.00^a^	2123.33^b^	52.600	0.013
	42	3124.77^ab^	3238.94^a^	2948.18^b^	67.000	0.010
BWG, g/bird/d	0–7	19.16^ab^	17.89^a^	20.87^b^	0.423	0.002
	7–14	38.94	41.28	38.76	0.883	0.080
	14–21	64.19	63.34	63.38	1.242	0.861
	21–28	82.72^ab^	85.77^a^	76.81^b^	2.340	0.026
	28–35	110.90^a^	117.00^a^	95.95^b^	4.070	0.001
	35–42	121.17	127.56	117.84	4.980	0.373
FI, g/bird/d	0–7	19.73^a^	19.42^a^	20.65^b^	0.249	0.002
	7–14	39.66	38.91	41.77	1.224	0.234
	14–21	118.72^a^	118.41^a^	114.08^b^	1.001	0.002
	21–28	129.19	129.09	129.38	0.260	0.744
	28–35	178.11	171.24	180.76	8.800	0.743
	35–42	216.46	200.05	218.48	14.290	0.630
FCR	0–7	1.03	1.08	1.03	0.018	0.051
	7–14	1.02	0.94	1.06	0.054	0.277
	14–21	1.86	1.88	1.81	0.033	0.282
	21–28	1.56	1.51	1.69	0.057	0.160
	28–35	1.58	1.47	1.90	0.136	0.145
	35–42	1.79	1.57	1.85	0.126	0.317
Overall performance	0–21 BWG, g/bird/d	41.14	40.88	40.81	0.643	0.932
	0–21 FI, g/bird/d	59.19	58.84	58.84	0.685	0.869
	0–21 FCR	1.46	1.44	1.44	0.021	0.821
	22–42 BWG, g/bird/d	105.99^a^	110.11^a^	96.86^b^	2.344	0.001
	22–42 FI, g/bird/d	174.59	166.79	176.21	7.450	0.654
	22–42 FCR	1.66	1.52	1.82	0.104	0.194
	0–21 Mortality, %	6.00	2.00	5.00	2.030	0.356
	22–42 Mortality, %	3.33	0.00	0.00	1.900	0.999

BWG = Body weight gain; Pooled SEM = Pooled standard error of the mean. Means in the same row with different letters differ significantly (*P* ≤ 0.05).

**Table 3:** Effect of dietary pure FA and CPE on broiler carcass traits (%) relative to BW.

Parameter *N* = 10	Control	FA 100 mg/kg	CPE 600 mg/kg	Pooled SEM	*P*-value
**Age: 21 d**					
Intestine	6.33	6.35	6.68	0.14	0.489
Liver	2.97	3.02	3.12	0.06	0.649
Spleen	0.12	0.11	0.13	0.01	0.517
Bursa of Fabricius	0.24	0.23	0.28	0.01	0.152
Gizzard	3.25	2.90	3.28	0.09	0.140
Heart	0.63	0.62	0.66	0.01	0.348
**Age: 42 d**					
Intestine	4.08^b^	4.06^b^	4.60^a^	0.09	0.009
Liver	2.21	2.28	2.34	0.04	0.468
Spleen	0.13	0.13	0.13	0.01	0.991
Bursa of Fabricius	0.20	0.20	0.19	0.01	0.959
Gizzard	2.14	2.21	2.34	0.06	0.343
Heart	0.52	0.48	0.53	0.01	0.126

FA: Ferulic acid; CPE: Corn pericarp extract (600 mg/kg; FA-equivalent: 100 mg/kg). Pooled SEM = Pooled standard error of the mean. Means in the same row with different letters differ significantly (*P* ≤ 0.05).

**Table 4:** Effect of dietary pure FA and CPE on plasma lipid profiles.

Parameter	Control	FA 100 mg/kg	CPE 600 mg/kg	Pooled SEM	*P*-value
**Age: 21 d**					
Total cholesterol, mg/dl	126.73	132.40	125.12	4.98	0.825
Total triglycerides, mg/dl	37.70	37.20	40.30	3.76	0.941
NEFA, mmol/L	182.33	184.61	119.32	11.77	0.094
**Age: 42 d**					
Total cholesterol, mg/dl	129.87	169.99	156.45	6.27	0.056
Total triglycerides, mg/dl	59.49	66.34	66.09	4.51	0.789
NEFA, mmol/L	130.78	176.78	112.65	12.50	0.125

FA: Ferulic acid; CPE: Corn pericarp extract (600 mg/kg; FA-equivalent: 100 mg/kg). Pooled SEM = Pooled standard error of the mean.

## Data Availability

The data that support the findings of this study are available at the National Center for Biotechnology Information (NCBI) Sequence Read Archive (SRA) BioProject ID PRJNA1294991.
