# Supplementary material for: Dietary supplementation with pure or plant-derived phytochemicals alters growth performance, lipid profiles, hepatic gene expression, and intestinal health in broiler chickens
Source: J Appl Poult Res. Author manuscript; Available in PMC 2026 Jul 8. (PMC13341142; doi:10.1016/j.japr.2026.100683)
Supplement: 1 [file NIHMS2180496-supplement-1.docx]

**Supplemental Table 1. List of primers used for qRT-PCR analysis.**

| Gene | Primer sequence (5’ to 3’) | Orientation |
| --- | --- | --- |
| *IL10* | GCTGAGGGTGAAGTTTGAGG  ATGCTGTGCTGATGACTGGT | Forward  Reverse |
| *IL6* | TGTGCAAGAAGTTCACCGTG  ACTCGACGTTCTGCTTTTCG | Forward  Reverse |
| *INFγ* | TCAAAGCCGCACATCAAACA GAAGAGTTCATTCGCGGCTT | Forward  Reverse |
| *GPX1* | GATGTTCGAGAAGTGCGAGG  AAGTTCCAGGAGACGTCGTT | Forward  Reverse |
| *GRX* | GTGACGGTGA CATCCTCTGA  AGATCCCCTTTCTGGTGGTG | Forward  Reverse |
| *GST* | CACTATGCCAACACACGAGC  GGGCTCTCTCCTTCAGATCC | Forward  Reverse |
| *SOD1* | ATTACCGGCTTGTCTGATGG  CCTCCCTTTGCAGTCACATT | Forward  Reverse |
| *GAPDH* | CTGTTGTTGACCTGACCTGC  TCAAAGGTGGAGGAATGGCT | Forward  Reverse |
